# Phytochemical Composition and Multifunctional Applications of *Ricinus communis* L.: Insights into Therapeutic, Pharmacological, and Industrial Potential

**DOI:** 10.3390/molecules30153214

**Published:** 2025-07-31

**Authors:** Tokologo Prudence Ramothloa, Nqobile Monate Mkolo, Mmei Cheryl Motshudi, Mukhethwa Michael Mphephu, Mmamudi Anna Makhafola, Clarissa Marcelle Naidoo

**Affiliations:** Department of Biology and Environmental Sciences, School of Science and Technology, Sefako Makgatho Health Science University, Pretoria 0204, South Africa; ramothloatokologo@gmail.com (T.P.R.); nqobile.mkolo@smu.ac.za (N.M.M.); cheryl.motshudi@smu.ac.za (M.C.M.); 201429242@swave.smu.ac.za (M.M.M.); annammamudimakhafola@gmail.com (M.A.M.)

**Keywords:** *Ricinus communis*, ricin toxicity, chemical composition, pharmacological activity, traditional uses, industrial applications, botanical features, castor oil

## Abstract

*Ricinus communis* (Euphorbiaceae), commonly known as the castor oil plant, is prized for its versatile applications in medicine, industry, and agriculture. It features large, deeply lobed leaves with vibrant colours, robust stems with anthocyanin pigments, and extensive root systems for nutrient absorption. Its terminal panicle-like inflorescences bear monoecious flowers, and its seeds are enclosed in prickly capsules. Throughout its various parts, *R. communis* harbours a diverse array of bioactive compounds. Leaves contain tannins, which exhibit astringent and antimicrobial properties, and alkaloids like ricinine, known for anti-inflammatory properties, as well as flavonoids like rutin, offering antioxidant and antibacterial properties. Roots contain ellagitannins, lupeol, and indole-3-acetic acid, known for anti-inflammatory and liver-protective effects. Seeds are renowned for ricin, ricinine, and phenolic compounds crucial for industrial applications such as biodegradable polymers. Pharmacologically, it demonstrates antioxidant effects from flavonoids and tannins, confirmed through minimum inhibitory concentration (MIC) assays for antibacterial activity. It shows potential in managing diabetes via insulin signalling pathways and exhibits anti-inflammatory properties by activating nuclear factor erythroid 2-related factor 2 (Nrf2). Additionally, it has anti-fertility effects and potential anticancer activity against cancer stem cells. This review aims to summarize *Ricinus communis*’s botanical properties, therapeutic uses, chemical composition, pharmacological effects, and industrial applications. Integrating the current knowledge offers insights into future research directions, emphasizing the plant’s diverse roles in agriculture, medicine, and industry.

## 1. Introduction

The castor bean plant, or *Ricinus communis*, is a significant member of the Euphorbiaceae family, native to tropical and subtropical regions [1]. It showcases remarkable phenotypic plasticity, with diverse morphological traits, such as lobed leaves and a deep taproot system [1]. The castor bean plant seeds are a vital source of essential oil seeds, primarily cultivated in South America, Africa, and India [2]. Extensive research has been conducted on this plant, underscoring its diverse chemical composition and wide-ranging applications [3]. Studies have revealed that the plant’s leaves contain phytochemicals that can combat various infections by acting as antibacterial and antifungal agents [4]. The plant’s potential for environmental remediation has also been explored, particularly its effectiveness in handling soils contaminated with heavy metals [5]. *Ricinus communis* is chemically constituted of biologically active molecules like ricinoleic acid, which is a principal fatty acid in castor oil possessing anti-inflammatory activity; ricinine, which is an alkaloid possessing insecticidal and putative anticonvulsant activities; rutin, which is a flavonoid with antioxidant activity; lupeol, which is a triterpenoid with anti-inflammatory and anticancer activities; and tannins, which are polyphenolic compounds possessing antimicrobial and astringent activities [6]. These molecules are referred to in this manuscript below with respect to their therapeutic and toxicological activities.

Moreover, research has delved into the plant’s toxic properties, evaluating its genotoxic effects and toxicity against insect vectors [7,8]. The plant has been harnessed to produce gold nanoparticles, showcasing its versatility in material science and nanotechnology [9]. Studies have also focused on using castor bean oil in biodiesel production, emphasising the optimisation of processing parameters [2]. Additionally, the plant’s pharmacological and toxicological effects on mammalian cells have been investigated, providing insights into its potential therapeutic uses [10]. The seeds contain various compounds, with ricinoleic acid being a prominent feature in the extracted castor oil; in contrast, the seed coat houses the well-known protein ricin, showcasing the plant’s multifaceted applications [11].

Comprehensive studies have revealed variations in chemical profiles across different cultivars and plant parts, presenting a rich landscape for exploration [3]. The castor bean plant’s chemical complexity has spurred research into its industrial applications and medicinal properties [12,13]. Historically, *R. communis* has left an indelible mark on traditional medicine, featuring prominently in medieval practices [14]. The plant’s applications have evolved, culminating in its recognition as an essential oil seed crop with vast agronomic importance [3]. The adaptability of different cultivars, with varying maturation cycles and stress tolerances, underscores its relevance to diverse agricultural practices [15]. The potential of *R. communis* as a bioresource has stimulated a broad spectrum of studies, delving into diverse facets, from seed maturation and plant development to responses to biotic and abiotic stresses [16,17].

The captivating blend of historical significance, botanical diversity, and chemical complexity converges in the medicinal realm, where the oil extracted from *R. communis* seeds has demonstrated pharmacological promise [5,18]. Scarpa and Guerci’s pioneering study [19] laid the foundation for understanding the medicinal uses of *R. communis*, revealing its global prevalence in traditional medicine. However, as the authors suggested, a deeper exploration into the phytochemical and pharmacological aspects was warranted to unravel the compounds responsible for the plant’s medicinal properties [19]. This call for further investigation has since spurred a plethora of research dedicated to unravelling the chemical composition and pharmacological activities of *R. communis* extracts and isolated compounds.

This literature review aims to unravel the complexities of *R. communis*, comprising its botanical features, medicinal significance, chemical composition, and pharmacological activities. By examining the diverse studies that span traditional medicinal uses, industrial applications, and innovative biotechnological research, the aim is to provide a holistic understanding of this enigmatic plant. Through synthesising the existing knowledge, this review seeks to illuminate the pathways for future research, fostering a deeperappreciation for the multifaceted nature of *R. communis* and its pivotal role in agriculture, medicine, and industry.

## 2. Methodology

This review was carried out by systematically searching the literature in scientific databases such as PubMed, Scopus, ScienceDirect, and Google Scholar. The keywords used for the search were “*Ricinus communis*”, “ricin toxicity”, “chemical composition”, “pharmacological activity”, “traditional uses”, “industrial applications,” and “botanical features.” Boolean operators “AND” and “OR” were used to restrict the searches. The papers were chosen based on relevance, recency (2010–2023), and scientific merit. Duplicate studies and articles that were not in the English language were excluded.

Following the PRISMA 2020 (Preferred Reporting Items for Systematic Reviews and Meta-Analyses) standards, a systematic literature search was conducted to find papers relevant to *R. communis*. The search covered publications from 1980 to 2024, ensuring a comprehensive historical and contemporary overview of the scientific discourse on the species. The identification phase involved searching on four major electronic databases, namely, PubMed (*n* = 450), Scopus (*n* = 350), Web of Science (*n* = 300), and Google Scholar (*n* = 150), resulting in 1250 records. An initial screening process removed 200 duplicate entries and 180 studies that did not directly pertain to *R. communis*, leaving 620 unique records for title and abstract screening. The full texts of 620 articles were subsequently evaluated for eligibility. A total of 161 studies were excluded for the following reasons: irrelevance to the research question (*n* = 110), inappropriate study design (*n* = 45), and language limitations (articles not published in English; *n* = 6).

**Figure 1:**
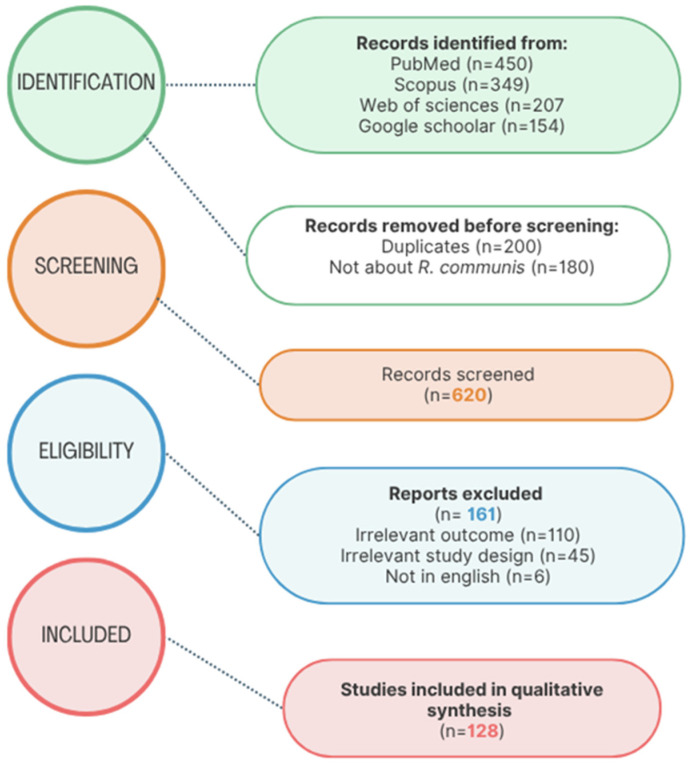
PRISMA flow diagram illustrating the systematic process of identification, screening, eligibility assessment, and inclusion of studies in the literature review.

Ultimately, 129 studies met all inclusion criteria and were incorporated into the qualitative synthesis. These studies form the empirical and conceptual foundation of this literature review and are represented in the accompanying PRISMA flow diagram in Figure 1 below.

## 3. Taxonomy and Botanical Features

### 3.1. Euphorbiaceae Family

According to Rahman et al. [20], the Euphorbiaceae family, commonly called the spurge family, is a diversified collection of plants with a wide range of species that show exceptional adaptation across numerous ecological niches. The family’s morphological diversity, which ranges from tiny herbs to enormous trees, reflects this adaptability and illustrates the family’s evolutionary flexibility [20]. One of the most extensive and varied families of flowering plants, the Euphorbiaceae family, usually referred to as the spurge family, is widely distributed throughout the tropics and comprises more than 300 genera and 8000 species [21]. The Euphorbiaceae family is well known for its ethnomedicinal applications and for producing bioactive diterpenoids, which have been linked to the biological activity of numerous species [22]. A 5/7/6-tricyclic ring system with poly-hydroxyl groups characterises the naturally occurring daphnane diterpenoids, which are primarily found in plants belonging to the Thymelaeaceae and Euphorbiaceae families [22]. Certain unique types also possess a distinctive orthoester motif triaxially connected at carbon positions [23].

A few of the 6300 species and 219 genera in the Euphorbiaceae family are native to Brazil and are found in tropical and subtropical climates [24]. Moreover, with 6745 species and 218 genera, the Euphorbiaceae family of angiosperms is one of the most taxonomically complicated and varied [25]. The family is renowned for its abundant rubber, oil, timber, starch, medicines, and other plant items vital to the economy [26]. Naki et al. [27] further mentioned that the Euphorbiaceae family is widely distributed throughout Indonesia and has long been utilised experimentally in medicine.

**Figure 2:**
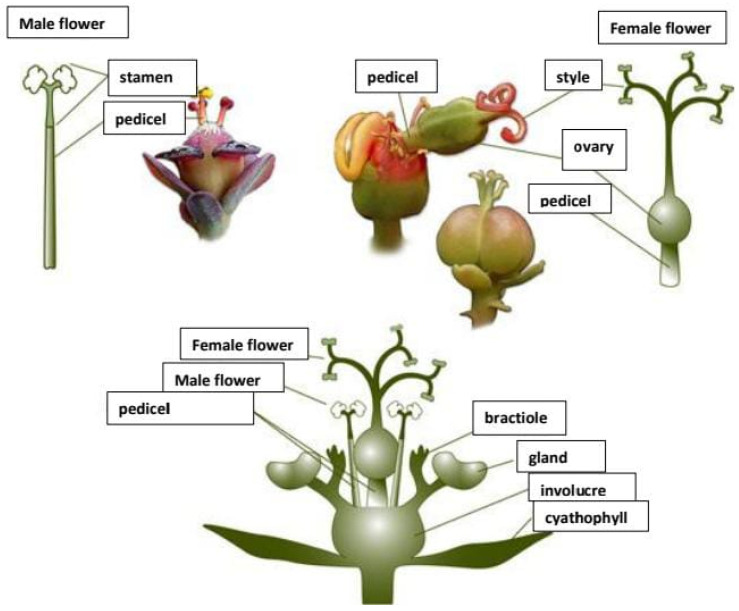
Diagrammatic representation of Cyanthia of the genera *Euphorbia* [34].

The Euphorbiaceae family is recognised as the most prevalent in some forest regions, such as the Congo Basin and the Amazon Rainforest [28]. Rich and varied flora, including *Manihot esculenta* (cassava) and *Jatropha curcas* (Barbados nut), which are abundant in both small-scale mined and unmined areas, are further characteristics of the Euphorbiaceae family that attest to its adaptation and resilience [29]. Furthermore, a few of the Euphorbiaceae family members are well known for producing latex, a liquid that acts as a defence against pests and herbivores [30]. One prominent example is the rubber tree (*Hevea brasiliensis*), which yields economically valuable latex in natural rubber manufacturing [20]. The family also includes simple but highly variable leaves, and small, usually unisexual or bisexual flowers frequently gathered in inflorescences [31]. In contrast to many other plant families, the Euphorbiaceae have structures in genera such as *Euphorbia* called cyathia, depicted in Figure 2, which are composites of several flowers that resemble a single flower [32]. According to Lopes et al. [33], the fruits of Euphorbiaceae plants are often capsules that can rupture and release seeds over a long distance, providing a broader seed dispersion for the species’ survival and propagation.

#### 3.1.1. Taxonomy of *R. communis*

According to Chouhan et al. [35], the castor oil plant is classified as follows: Kingdom: Plantae, Phylum: Spermatophyta, Subphylum: Angiospermae, Class: Dicotyledonae, Order: Euphorbiales, Family: Euphorbiaceae, Genus: *Ricinus*, Species: *communis*.

#### 3.1.2. Botanical Features of *Ricinus communis*

##### Leaves

**Figure 3:**
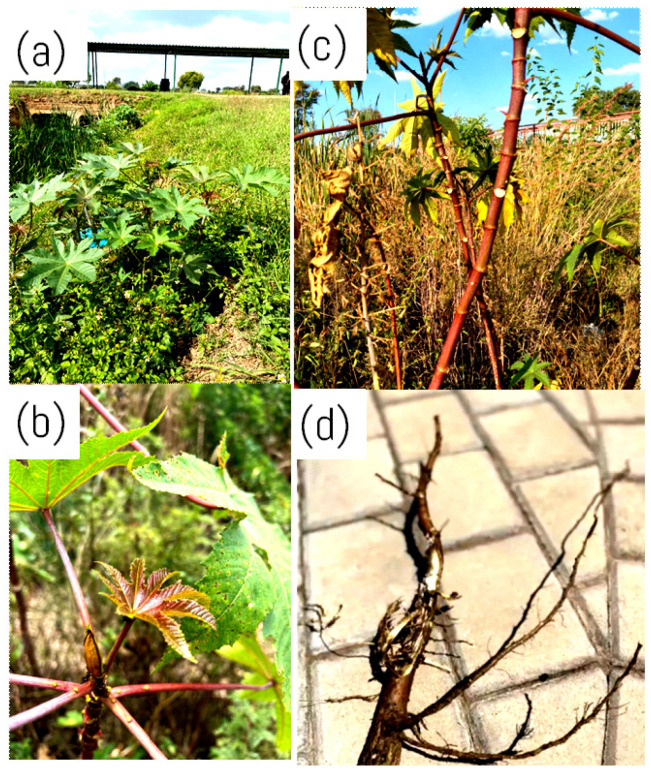
Different *Ricinus communis* plant parts growing wild in its natural habitat at the Sefako Makgatho Health Sciences University campus (25.6192° S, 28.0161° E). (**a**) Shrub; (**b**) stem; (**c**) young leaf; (**d**) roots. Image captured by T.P. Ramothloa.

The leaves of *R. communis* have a firm texture and glossy green hue and are relatively large, as seen in Figure 3a [36]. These leaves can grow to a diameter of 60 centimetres and are deeply split, with palmately lobed surfaces [36]. The ornamental attractiveness of the leaves is attributed to their usual five to eleven-pointed lobes that radiate from a central point and have serrated borders [37]. According to Suurbaar et al. [4], the veins of the leaves are visible and run outward from the central midrib to the tips of the lobes, where they aid in the movement of nutrients and provide structural support. The leaves can range from vivid green to deep red, depending on the anthocyanin pigments present [38]. As the castor oil plant leaves develop, they change from a dark reddish-purple or bronze shade to a dark green one, as seen in Figure 3b [38]. The observed shades in the leaves are influenced by the different stages of their development and are caused by the variable levels of anthocyanin colouring present in the leaves [38].

##### Stem

*Ricinus communis* has a strong stem and smooth surface, essential for maintaining the plant’s overall structure [31]. The stem grows vertically and branches into a distinct structure that supports the plant’s stability and resilience in various environmental circumstances [31]. According to the literature, *R. communis* has a robust, erect, and succulent stem that can grow up to 1–2 m in height [39]. The stem is typically smooth, hairless, and light green, but some cultivars may have reddish or purple stems due to anthocyanin pigments depicted in Figure 3c [40]. These anthocyanin pigments are water-soluble pigments belonging to the flavonoid class of compounds responsible for the red, purple, and blue hues in various fruits, flowers, and plant stems [41]. According to Li et al. [42], these plant pigments have several purposes, such as attracting pollinators, protecting plants from UV rays, and fending off herbivores. The stem has prominent leaf scars and is covered by a waxy cuticle with a white, milky sap inside, also known as latex [43]. It is hollow inside, with a central pith surrounded by a ring of vascular bundles composed of xylem and phloem tissues that transport water and nutrients between the roots and the rest of the plant [44].

##### Roots

*Ricinus communis* roots are thick and fleshy and can grow up to 2 m deep into the ground [35]. The roots primarily comprise principal and secondary roots, with some adventitious roots emerging from the stem [45]. The primary roots are thick and gradually taper, forming a taproot system that aids the plant’s anchoring and access to profound soil moisture [35]. Secondary roots are thin and fibrous, branching out laterally from the primary roots to form a dense network that absorbs moisture and nutrients from the soil [45]. The roots have a thin layer of root hairs that maximise the surface area for moisture and nutrient absorption, depicted in Figure 3d [35].

##### Flowers and Inflorescences

**Figure 4:**
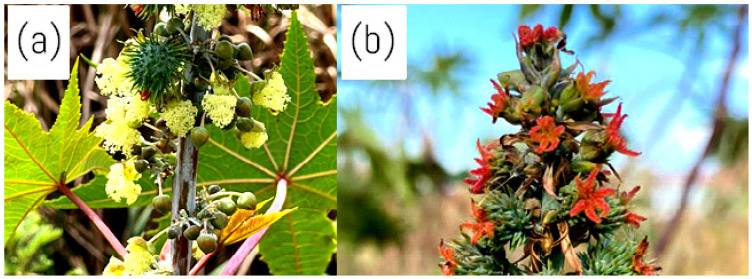
*Ricinus communis* flowers from a plant in its natural habitat at Sefako Makgatho Health Sciences University. (**a**) The male flower. (**b**) female flower. Image captured by T.P. Ramothloa.

*Ricinus communis* has a terminal panicle-like inflorescence of closely spaced clusters of tiny, monoecious flowers [31]. On the same plant, *R. communis* yields both male and female flowers [46]. However, the female flowers are usually at the top of the inflorescence, and the male flowers are at the bottom [46]. The flowers are produced in terminal racemes or panicles, including male and female ones [47]. The male flowers are relatively large and greenish, with a three-lobed calyx, a three-lobed ovary, and a single style with three stigmas, depicted in Figure 4a [46]. The female flowers are small and reddish, with a five-lobed calyx, a five-petaled corolla, and numerous stamens depicted in Figure 4b [47]. Since the flowers are protandrous, the male flowers reach maturity before the female flowers, which assists in cross-pollination [46]. This unusual flower arrangement plays a vital role in developing recognisable castor oil seeds and aiding the plant’s reproductive strategy [46].

##### Seeds

**Figure 5:**
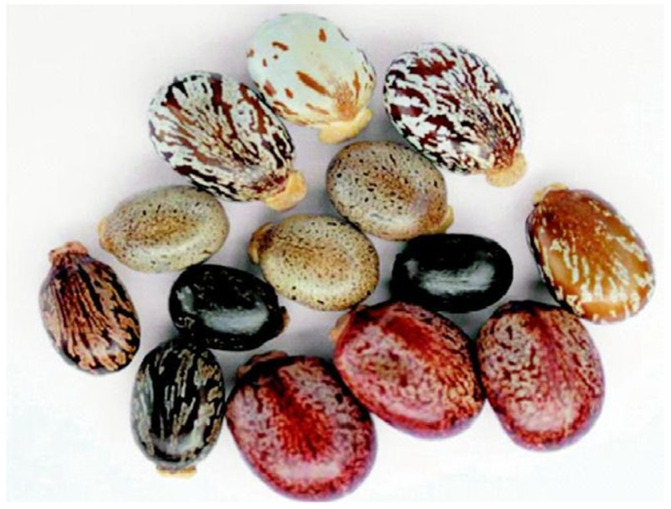
Variations in size and colour of *Ricinus communis* seeds [53].

*Ricinus communis* seeds have complex morphological characteristics that add to their uniqueness and ecological significance [48]. The seeds are firm and glossy, with colours ranging from light brown to dark reddish-brown, and they are enclosed in a spiky, spherical fruit capsule [49]. These seeds have an oval shape and a smooth, glossy surface; they are relatively large, usually ranging from one to two centimetres [1]. Their rigid and resilient exterior integument acts as a buffer, preventing outside forces and environmental stressors from wounding the developing plant inside [50]. When examined more closely, the seeds exhibit distinctive marks and carvings that enhance their visual attractiveness in addition to complex patterns and textures (Figure 5) [48]. According to Herawati et al. [51], the seeds’ surface may display detailed differences in texture that are indicative of both underlying genetic variety and environmental factors. Additionally, because of their sturdy construction and appealing look, which attract a variety of seed dispersers like birds and mammals, the seeds serve a crucial ecological function in seed dispersal mechanisms [52].

#### 3.1.3. Geographical Distribution of *Ricinus communis*

*Ricinus communis* is native to the Indian subcontinent, Eastern Africa, and the Southeast Mediterranean Basin [54]. However, it has been widely cultivated and naturalized in many other regions, including tropical and subtropical areas such as the Americas and China, and throughout all the continents except Antarctica [31]. Its seeds were valued for their industrial and therapeutic qualities in ancient civilisations [14]. Rana et al. [14] shed light on the plant’s traditional usage and pharmaceutical potential, highlighting its complex value. Due to the plant’s adaptation to warm, sunny climates and well-drained soil, it has historically been cultivated in these areas, which has helped it become a profitable crop [31]. Through various factors, including human trade and accidental seed spread, *R. communis*’s global distribution surpassed its original range [31]. The distribution of *R. communis* in South Africa is influenced by various factors, including its historical origins, ecological adaptability, and human activities [55]. The plant’s distribution in South Africa is closely linked to its adaptability to diverse environmental conditions, including well-drained soils and warm temperatures [56]. The plant’s historical uses in traditional medicine and its economic significance have also contributed to its intentional cultivation and spread within the country [57]. Furthermore, *R. communis* in South Africa raises concerns about its potential invasiveness and ecological impacts on native vegetation and ecosystems [58].

South Africa’s provinces show a varied distribution of *R. communis* as it can survive in various environments, from dry inland regions to coastal locations [59]. It is found in the fynbos biome of the Western Cape, especially in places with well-drained soils and moderate rainfall [59]. It also spreads to the Eastern Cape, taking over scrublands and grasslands [60]. This species, adapted to extreme environmental circumstances, is also common in semi-desert parts of the Northern Cape [61]. Moreover, it is also found in disturbed places like roadside ditches and uninhabited fields in Gauteng [62]. Its frequent occurrence in savanna and forest habitats in the provinces of Limpopo and Mpumalanga demonstrates its capacity to flourish in various ecosystems [59,63]. Additionally, it exists in the KwaZulu-Natal (KZN) province in the riverbank and coastal regions where there is relatively more precipitation [59,63]. This distribution pattern highlights the ecological adaptability of *R. communis*, which enables it to endure and proliferate in various South African settings [64]. Table 1 summarises the distribution of *R. communis* globally, emphasising the climate and type of soil found in the area, while Table 2 summarises the distribution of *R. communis* in South Africa, also emphasising the climate and the soil type found in the area, as well as the habitat of the plant.

## 4. Traditional Applications

*Ricinus communis* has many uses that demonstrate the symbiotic interaction between humans and the natural environment and show its historical significance and ongoing relevance in modern contexts [20]. The customary applications of *R. communis* are profoundly embedded throughout a wide range of global civilisations [14]. The seeds have long been used mainly for castor oil production, and then the oil was used as a strong laxative that often assisted in relieving constipation and encouraging regular bowel movements [76]. According to Suurbaar et al. [4], castor oil has also been included in several topical skincare formulations due to its moisturising and antibacterial qualities, which make it useful in the treatment of dermatological diseases like dermatitis, eczema, and acne. Castor oil is highly valued for its skin-cleansing and rejuvenating properties in traditional medical systems such as Ayurveda and traditional Chinese medicine; hence, it is commonly used in herbal medicines and cosmetic goods [77].

Additionally, various parts of the plant offer specific therapeutic benefits. The leaves act as an insecticide against aphids, rust mites, mosquitoes, and whiteflies, and are used to increase milk production in cattle and nursing mothers [78]. They also serve medicinal purposes, such as relieving stomach aches and treating eye infections and narcotic poisoning [78]. Using hot water extracts, the roots are valued for their efficacy in treating toothaches and jaundice [78]. *Ricinus communis* is a multipurpose plant in agricultural settings, as its allelopathic properties help deter pests and promote biodiversity in agroecosystems [3]. Its rapid growth and resilience make it suitable for soil conservation efforts [10]. Additionally, *R. communis* is culturally significant in various societies’ folklore, mythology, and traditional rituals, as it is associated with purification and protection against evil spirits [11].

In certain regions of South America, castor oil is traditionally used as an antiparasitic drug, primarily for the expulsion of intestinal worms. Brazilian and Colombian ethnomedicinal applications involve oral intake of small amounts of warm castor oil to cure helminthic infections, with activity noted against *Strongyloides*, tapeworms, and roundworms [79]. This folk use is supported by in vitro pharmacological studies, which showed that castor oil is cysticidal against *Entamoeba histolytica* and other gastrointestinal parasites [80]. Table 3 summarises the traditional uses of *R. communis* for treating various diseases or ailments.

## 5. Chemical Composition

The chemical composition provides information about the molecular makeup of an item or organism by describing the types and arrangements of chemical elements and compounds that are present [3]. Conversely, active constituents are substances found in a natural product or organism with pharmacological or physiological effects [83]. *R. communis* is a chemically complex plant, with each anatomical part consisting of distinct sets of bioactive constituents that add to its overall pharmacological, therapeutic, and toxicological properties [3]. Phytochemical constituents found in the leaves, seeds, roots, stems, and flowers of the plant not only explain its medicinal importance but also identify its ecological standing [2].

The leaves of *R. communis* have drawn extensive studies on their phytochemical constituents, revealing the occurrence of prominent secondary metabolites such as tannins, saponins, alkaloids, and flavonoids [84]. These phytochemicals are commonly renowned for their medicinal significance, particularly due to their antioxidant, antimicrobial, and anti-inflammatory activities [85]. Phytochemical analysis of the leaf extracts was consistent with the presence of selected phenolic acids, such as gallic acid, ellagic acid, and quercetin, and flavonoids, such as rutin and epicatechin [86]. The co-presence of these bioactive compounds validates the therapeutic significance of the leaves to traditional and modern phytotherapeutic practices.

Similarly, the seeds of *R. Communis* possess a stable and intricate chemical makeup that serves as the basis for most of the plant’s pharmaceutical and industrial uses. Alkaloids like ricinine, flavonoids, saponins, and phenolic acids are found in the seeds [11,86]. These phytochemicals are not just implicated in the well-known anti-inflammatory and anticancer activities of the plant but also its toxicity, as exemplified by the ricin content, a highly toxic protein toxin that has also been extracted from the seeds [2]. This two-sidedness of the compounds makes the seed extracts a subject of considerable interest in both pharmacological and toxicological research. Besides the leaves and seeds, the roots of *R. communis* contain numerous bioactive components. Among them is lupeol, a triterpenoid with well-established anti-inflammatory and anticancer activities [5]. The roots are also discovered to contain indole-3-acetic acid, a plant growth regulator that participates in the biosynthesis of other secondary metabolites, which functions to enhance the plant’s versatility and provide potential drug applications [5]. While not as well-documented as the seeds or leaves, these compounds in the root contribute to the plant’s overall medicinal value.

The *R. communis* chemical profile is also found in stems and flowers, though the studies are less extensive. Available data report the presence of tannins, which have antioxidant activity, and β-caryophyllene, a sesquiterpenoid with anti-inflammatory activity and plant defence properties [11]. Also, ricinine, present in seeds, was found to be present in stems, playing a role in plant defence chemicals [11].

**Figure 6:**
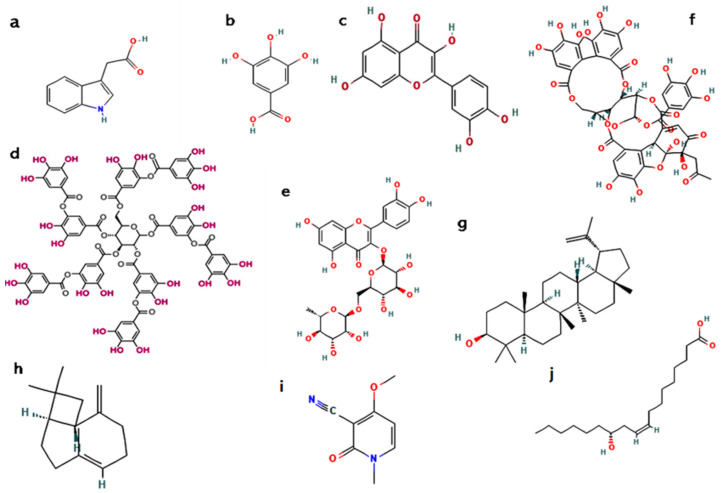
Chemical structures of active constituents found in various parts of the *Ricinus communis* plant: (**a**) indole-3-acetic acid; (**b**) gallic acid; (**c**) quercetin; (**d**) tannic acid, (**e**) rutin, (**f**) ellagitannins; (**g**) lupeol; (**h**) β-caryophyllene; (**i**) ricinine; (**j**) ricinoleic acid.

The *R. communis* flowers, although less studied, are also associated with their pharmacological activity. Floral tissues were found to possess flavonoids such as rutin, which are both recognised for their vascular protective and antioxidant activity [3]. This suggests that even the lesser studied components of the plant may be of therapeutic significance that should be investigated. Collectively, *R. communis* shows a chemically intricate and pharmacologically diverse profile for all its components. Figure 6 depicts the chemical structures of some of the active constituents found in the various parts of the plant. Table 4 summarises the chemical compounds in different plant parts and their most abundant active constituents.

## 6. Pharmacological Activity

Pharmacological activities include a variety of actions that substances, especially medications or other medical compounds, take on biological systems [88]. These activities may have therapeutic benefits, such as reducing symptoms, preventing illness, or enhancing well-being, in addition to possible negative consequences or reactions [88]. When discussing medicinal plants, the term “pharmacological activities” refers to the range of biological effects that these plants’ bioactive chemicals display [76]. *Ricinus communis* is well known for its pharmacological properties, mostly linked to its seeds [89]. *Ricinus communis* seeds yield castor oil, traditionally used for its therapeutic benefits, and ricin, a poisonous protein [89].

### 6.1. Antioxidant Activity

The discovery of the antioxidant activity of *R. communis* has been intensively improved by several investigations [90,91]. To evaluate the antioxidant potential of *R. communis* roots, various solvent extracts (methanol, ethanol, ethyl acetate, n-hexane, chloroform, n-butanol, and aqueous) were prepared and analysed using standard antioxidant assays. Ahmed and Iqbal [90] investigated these extracts for their ability to scavenge DPPH radicals and measured their total phenolic content (TPC) and total flavonoid content (TFC). The results indicated significant antioxidant activity across all extracts, with ethyl acetate and chloroform showing the highest DPPH scavenging activity at 41%. Additionally, the TPC analysis revealed notable activity in aqueous, n-butanol, and ethyl acetate extracts, with values of 131 mg/mL, 127 mg/mL, and 117 mg/mL gallic acid equivalent, respectively. Regarding TFC, methanol and aqueous extracts demonstrated the highest activity at 32 µg/mL each, compared to 38 µg/mL in the control. These findings highlight *R. communis* roots as a powerful antioxidant source, implying that they could be useful in medicinal applications.

In a related study, Ihekuna et al. [91] investigated the antioxidant activity of ethanolic extracts from *R. communis* seeds and leaves. In both in vivo and in vitro assessments, *R. communis* extracts displayed potent antioxidant activity, with IC_50_ values of 77.17 ± 0.36 and 69.33 ± 0.26 μg/mL for seed and leaf extracts in DPPH assays and 62.86 ± 0.54 and 62.39 ± 0.18 μg/mL in nitric oxide radical inhibition assays, respectively. Treatment with these extracts boosted catalase and superoxide dismutase (SOD) levels in the liver and kidneys, suggesting a protective effect against oxidative stress. According to their findings, the ethanolic extracts had a substantial amount of antioxidant activity, suggesting the presence of bioactive substances that could reduce oxidative damage and neutralise free radicals.

Tuyen et al. [85] investigated the antioxidant qualities of *R. communis* leaves. Using spectrophotometry and chromatography, the researchers found many phenolic chemicals in the leaves, such as flavanol glycosides, aromatic acids, and coumarinolignan. The compounds identified included cleomiscosin A, kaempferol-3-O-β-D-glucopyranoside, kaempferol-3-O-β-D-xylopyranoside, gallic acid, and vanillic acid. Cleomiscosin A was discovered for the first time in the *Ricinus* genus and had low DPPH radical scavenging activity (SC_50_ = 403.23 μg/mL). The presence of these compounds in *R. communis* leaves emphasises the plant’s ability to scavenge free radicals and guard against oxidative stress, as these compounds are known for their antioxidant properties.

The studies mentioned above used solvents of varying polarity, such as ethyl acetate, chloroform, methanol, and ethanol, which likely influenced the antioxidant activities observed by selectively extracting different phytochemicals. This methodological variation suggests a lack of standardization that complicates direct comparison between studies, though it also highlights the importance of solvent choice in maximizing bioactive compound yield. This variation in solvent polarity is crucial, as more polar solvents (like methanol and aqueous) are more effective at extracting hydrophilic compounds such as phenolics and flavonoids, while less polar solvents (like chloroform and hexane) may extract non-polar antioxidant constituents. By linking solvent polarity to the type of compounds extracted, it becomes clearer why different extracts demonstrated varying levels of antioxidant activity. While Ahmed and Iqbal [90] emphasised the correlation between total phenolic and flavonoid content and antioxidant activity, Tuyen et al. [85] identified specific phenolic compounds like cleomiscosin A with low activity, indicating that not all phenolics contribute equally to antioxidant potential. Nonetheless, all three studies consistently demonstrate that *R. communis* exhibits noteworthy antioxidant activity, supporting its potential application in oxidative stress-related therapies.

### 6.2. Antifungal/Antimicrobial Activity

*Ricinus communis*, known for its diverse pharmacological properties, has strong antifungal activity since its effectiveness against a range of fungal species has been demonstrated by research on its antifungal properties [92]. The antifungal potential of *R. communis* was investigated by Dikhoba et al. [92], who showed that the plant’s leaf extract was effective against fungi, with the minimum inhibitory concentration (MIC) ranging from 0.08 to 2.5 mg/mL, with *R. communis* leaf extract, among others, exhibiting promising antifungal activity. Additionally, *R. communis* had a MIC value of 0.08 mg/mL against *Fusarium verticilloides*, comparable to the antibiotic Amphoteracin B. Furthermore, the overall efficacy of *R. communis* against *Aspergillus ochraceous* was notable, indicating its potency in preventing fungal growth. To ascertain the MIC values or the concentration at which the growth of the fungal pathogen is stopped, the investigators used standard microbiological techniques.

Moreover, Suurbaar et al. [4] used assays such as disc diffusion, MIC, minimal lethal concentration (MLC), and minimum fungicidal concentration (MFC) methods to study the antibacterial and antifungal properties of *R. communis* leaf extracts. The solvent extracts from *R. communis*’s leaves displayed bacteriostatic and bactericidal effects against the tested organisms, with MIC values ranging from 3.13 to 25.0 mg/mL and MBCs from 200 to 400 mg/mL. For *Candida albicans*, MFCs fell between 200 and 400 mg/L, indicating potential antifungal activity. These findings affirm the antimicrobial properties of *R. communis*’s leaves and highlight the extractability of biologically relevant phytochemicals using aqueous, methanol, and ethanol solvents from its leaves.

Additionally, bimetallic nanoparticles made using *R. communis* leaf extracts have been shown to have antibacterial and antifungal activities by López-Ubaldo et al. [93]. The antibacterial activity of the nanoparticles was assessed using microbiological assays. The synthesised nanoparticles showed promising antibacterial activity against *Staphylococcus aureus*, *Escherichia coli*, and *Aspergillus niger*, with MIC concentrations ranging from 1.25 to 2.45 μg and MLC allowances from 2.45 to 9.81 μg.

Additionally, *R. communis* leaf extracts and fractions were studied by Istaufa et al. [94] for their antibacterial properties against various diseases, including tuberculosis (TB), which is caused by the bacteria *Mycobacterium tuberculosis*. The ethanolic extract showed the most promising results, completely inhibiting bacterial growth at 200 μg/mL, with an average colony count reduction from 20.33 at 100 μg/mL to 0 at 200 μg/mL. Other solvents, such as methanol and acetone, also exhibited inhibitory effects, with the methanol extract inhibiting growth at 100 μg/mL and reducing colonies from 17.33 at 100 μg/mL to 0 at 150 μg/mL. The acetone extracts reduced colony counts from 55.66 at 100 μg/mL to 0 at 200 μg/mL. The minimum inhibitory levels of various extracts were assessed, with n-hexane showing activity at 10,000 μg/mL, ethyl acetate at 40,000 μg/mL, and chloroform at 5000 μg/mL. These findings suggest that *Ricinus communis* L. leaf extracts have significant potential as a supplementary treatment for tuberculosis, effectively inhibiting *Mycobacterium tuberculosis* growth at various concentrations and solvents.

The different studies mentioned above used a variety of solvents, ranging from ethanol and methanol to chloroform and n-hexane, and techniques, like MIC, MLC, and disc diffusion, to assess the antifungal and antimicrobial properties of *R. communis*, with each method and solvent choice influencing the observed potency. While Suurbaar et al. [4] demonstrated strong antifungal activity using plant extracts, López-Ubaldo et al. [93] showed that the synthesis of bimetallic nanoparticles from *R. communis* could further enhance antimicrobial efficacy, suggesting a promising nanotechnology-based approach. Across all studies, *R. communis* consistently exhibited notable antifungal activity against pathogens like *Fusarium verticilloides*, *Aspergillus niger*, and *Candida albicans*, reinforcing its potential as a natural antifungal agent.

### 6.3. Antidiabetic Activity

*Ricinus communis* may have antidiabetic properties; recent research has explored this possibility, providing insight into the plant’s possible benefits [10]. In a study conducted by Benariba et al. [95], the objective was to ascertain the levels of total phenolic and flavonoid compounds in hydro-acetone extracts obtained from the roots of *R. communis* and the aerial parts of *Teucrium polium*. Additionally, the study aimed to evaluate these extracts’ antioxidant and antidiabetic properties using DPPH, FRAP, and α-amylase activity assays. Moreover, *T. polium* exhibited greater efficiency in inhibiting the α-amylase enzyme (EC_50_ = 924 ± 0.19 µg/mL) compared to *R. communis* extract (EC_50_ = 1300 ± 0.03 µg/mL) in the α-amylase activity assay. The study highlights the potential antidiabetic activity of *R. communis* extract, particularly its ability to inhibit α-amylase activity. The findings suggest that the hydro-acetone extract from the roots of *R. communis* may offer promising therapeutic benefits for managing diabetes or related metabolic disorders.

Additionally, Hajrah et al. [10] evaluated the antidiabetic effect of *R. communis* leaf extract using gene expression profiling in MCF7 cells. The results of the study showed that treatment with the extract at concentrations of 10 μg/mL and 50 μg/mL significantly increased the expression of PPAR-γ, a key regulator of glucose metabolism and fatty acid storage, by 2.5 and 3.2-fold, respectively, and PPARGC1A (PGC-1α), which interacts with PPAR-γ to regulate genes related to energy metabolism, by 1.8 and 2.4-fold, respectively. Furthermore, DPP4 expression was downregulated by 2.1 and 2.7-fold, respectively, in the extract. DPP4 is implicated in the breakdown of incretins, including GLP-1, which increases insulin production and lowers blood glucose levels. These results imply that phytochemicals included in *R. communis* leaf extract may be able to better manage diabetes by enhancing insulin resistance, lowering serum glucose levels, and enhancing glucose uptake in muscle and adipose tissue.

Moreover, the study conducted by Dayyih et al. [96] and colleagues investigated the influence of castor oil (CAO) on glycated haemoglobin (HbA1c) levels in rats with induced type 2 diabetes mellitus (T2D), particularly in comparison to the Sodium–Glucose Cotransporter 2 (SGLT2) inhibitor empagliflozin. Their research aimed to shed light on CAO’s potential as a therapeutic agent for diabetes management. HbA1c was utilised as a long-term glycaemic regulation predictor, with 6.5% or above indicating diabetes, 5.7–6.4% suggesting pre-diabetes, and less than 5.7% indicating health. Rats were divided into eight groups, including healthy and diabetic, and treated with CAO for various durations. Significant decreases in HbA1c were observed from the ninth week onwards in healthy and diabetic rats treated with CAO compared to non-treated groups (*p* < 0.05), indicating a reduction in blood sugar levels. Specifically, a decrease in HbA1c values was noted in healthy rats given CAO daily, whereas HbA1c levels increased in healthy rats without drug treatment. However, CAO alone did not achieve the same efficacy in lowering HbA1c levels as empagliflozin, a diabetes medication. Empagliflozin-treated rats showed a more significant decrease in HbA1c levels than CAO-treated rats, with an earlier onset of effect observed in the empagliflozin group (*p* < 0.05). The results of their study revealed a noteworthy finding regarding the impact of CAO on glycaemic control. This disparity suggests a differential effect of CAO in diabetic conditions, possibly due to the complex pathophysiology of diabetes. Despite its traditional use as a laxative, the study indicates that CAO may possess some antidiabetic activity, albeit less potent than conventional medications like empagliflozin.

The studies mentioned above investigating the antidiabetic potential of *R. communis* used a range of methodologies, including biochemical assays, gene expression profiling, and animal models with differing extract types and target mechanisms, which may account for variations in observed efficacy [10,95,96]. While Benariba et al. [95] demonstrated moderate α-amylase inhibition through hydro-acetone root extracts, Hajrah et al. [9] highlighted the molecular effects of leaf extract, showing the upregulation of genes like PPAR-γ and the downregulation of DPP4 involved in glucose metabolism. Dayyih et al. [96], using in vivo models, showed that castor oil led to modest reductions in HbA1c levels. However, it was less effective than standard SGLT2 inhibitors, suggesting that *R. communis* may contribute to glycaemic control but likely serves best as a complementary rather than primary therapy. Overall, the studies collectively indicate that *R. communis* possesses antidiabetic properties through enzymatic inhibition and gene-regulatory pathways, with potential for use in supportive diabetes management.

### 6.4. Anti-Inflammatory Activity

The anti-inflammatory qualities of *R. communis* have been highlighted by a recent study, suggesting that it may be helpful as a treatment for inflammatory ailments. To clarify the anti-inflammatory properties of *R. communis* leaves, Lee et al. [97] investigated how they might effectively activate Nrf2 to mitigate the effects of dexamethasone-induced muscular atrophy. The study evaluated muscle atrophy and Nrf2 activation using in vivo and in vitro models, offering mechanistic insights into the anti-inflammatory qualities of *R. communis* leaves. Rutin, identified as the predominant compound in *R. communis* with a content of 159.5 mg/100 g dry weight of extract, effectively prevented DEX-induced myotube atrophy in vitro, reducing muscle atrophy-related gene expression, proteasome activity, and intracellular reactive oxygen species (ROS) accumulation while improving mitochondrial function.

Additionally, *R. communis* leaf extract’s anti-inflammatory properties were also investigated by Hajrah et al. [10] using gene expression profiling in MCF7 cells. The results of the investigation showed that the TNFAIP6 gene (tumour necrosis factor-inducible gene 6) was upregulated by 2.3- and 3.1-fold, respectively, after treatment with the extract at doses of 10 μg/mL and 50 μg/mL. By reducing neutrophil infiltration in illness models, TNFAIP6, often referred to as TNF-stimulated gene 6 (TSG-6), plays a critical role in mediating anti-inflammatory and protective effects. The gene’s product aids in the anti-inflammatory response by binding to and interacting with several chemokines. These results suggest that the leaf extract of *R. communis* possesses the capacity to produce notable anti-inflammatory effects, which could potentially augment its medicinal advantages.

Furthermore, *R. communis* and *Withania somnifera* hydroalcoholic extracts were evaluated by Hussain et al. [98] for their anti-inflammatory properties. They showed potent antioxidant and anti-inflammatory properties both in vitro and in vivo. The study assessed the antioxidant and anti-inflammatory activities of *R. communis* leaf extract and *W. somnifera* extract. Both extracts demonstrated the dose-dependent inhibition of oedema in inflammation models. *Ricinus communis* extracts showed the maximum inhibition of ear oedema (51.49 ± 2.54%) and paw oedema (46.62 ± 8.98%) in comparison to *W. somnifera*, with *R. communis* also significantly inhibiting carrageenan-induced paw oedema (72.88 ± 13.79%). These effects were attributed to the extracts’ high phenolic and flavonoid contents. In comparison, *R. communis* displayed more promising antioxidant and anti-inflammatory effects than *W. somnifera*.

The studies mentioned above explored the anti-inflammatory activity of *R. communis* employing diverse models and analytical approaches, such as gene expression profiling, muscle atrophy assays, and inflammation-induced oedema models, leading to a broad understanding of its therapeutic potential [10,98]. Lee et al. [97] demonstrated that rutin-rich leaf extracts activated Nrf2 and countered dexamethasone-induced muscle atrophy by reducing oxidative stress and proteasome activity, while Hajrah et al. [10] reported the upregulation of the TNFAIP6 gene, which plays a central role in suppressing inflammation. Hussain et al. [98] further supported these findings by showing that *R. communis* extracts significantly reduced oedema in animal models, outperforming *W. somnifera* in anti-inflammatory and antioxidant effects. Overall, these studies confirm that *R. communis* exhibits strong anti-inflammatory activity through both gene-regulatory mechanisms and the phenolic compound-mediated inhibition of inflammation.

### 6.5. Anti-Fertility Activity

A study by McNeil et al. [99] investigated the short-term and long-term spermatogenic effects of a small *R. communis* seed variety. The n-hexane extract of the seeds was given intraperitoneally to male rats, and semen analysis was performed regularly. Spermatogenesis and sperm motility were shown to be suppressed for up to six weeks following therapy, according to the data. Microscopic analysis revealed normal appearance, liquefaction, and consistency of semen samples in all groups. However, within 72 h of treatment with the extract from *R. communis* seeds, semen parameters were significantly suppressed. The percentage of normal sperm forms decreased by 50% (from 80% in controls to 40% in treated groups), and motility decreased by 56% (compared to 88% in controls). The semen count was also markedly reduced to 49 × 10^6^ (compared to 99.5 × 10^6^ in controls). Over the following weeks, there was a gradual improvement in mean normal forms and motility, reaching 60% and 65%, respectively, by the sixth week, while semen count began recovering by the eighth week. These findings indicated that *Ricinus communis* seed extract had a reversible impact on semen parameters, suggesting its potential as a male contraceptive agent that requires further investigation. Thus, *R. communis* is shown to be a potent yet reversible anti-spermatogenic agent with notable anti-motility qualities, showing potential as a readily available and reasonably priced male contraceptive drug because of the plant’s origin.

A study by Desouky et al. [100] evaluated the impact of *Eucalyptus* oil and *R. communis* seed extract on the reproductive biology of *Theba pisana* snails using assays including egg laying capacity, hatching rates, histopathological analysis of hermaphrodite glands, antioxidant enzyme activities, and hormonal analysis. Results showed significant reductions in egg-laying capacity (*p* < 0.05) and hatching rates (*p* < 0.01) with both extracts, particularly with the *Ricinus* extract displaying a more pronounced effect. Additionally, histopathological analysis revealed disruption in the hermaphrodite glands of treated snails, indicating interference with spermatogenesis and oogenesis. Hormonal analysis demonstrated disturbances in reproductive hormones, including increased levels of luteinising hormone (*p* < 0.05), follicle-stimulating hormone (*p* < 0.01), and testosterone (*p* < 0.001). These findings suggest that both *Eucalyptus* oil and *Ricinus* extract adversely affect the reproductive physiology of *T. pisana*, with *Ricinus* extract exerting more substantial anti-fertility effects.

Another study by Iornmube et al. [101] investigated the contraceptive efficacy of *R. communis* minor (RICOM-1013-J) through oral administration in women volunteers. The results showed that a dosage range of 2.5–2.7 gm of RICOM-1013-J conferred protection against conception for 12 months. Sexual activity frequency was 2–3 times per week, and menstrual flow remained within typical limits throughout the study period. Examination of hormone profiles revealed notable differences between the treatment and control groups. Specifically, individuals in the treatment group exhibited significantly elevated levels of prolactin compared to those in the control group. Women administered RICOM-1013-J showed significantly elevated prolactin levels compared to the control group (mean: 1154.4 mIU/L vs. 171.5 mIU/L, *p* < 0.01). In contrast, luteinising hormone (LH) and follicle-stimulating hormone (FSH) levels varied across the groups (mean LH: 2.25 IU/L vs. 1.47 IU/L, mean FSH: 3.14 IU/L vs. 3.67 IU/L, *p* > 0.05). The findings suggest that RICOM-1013-J may induce alterations in hormone levels associated with contraceptive effects, warranting further investigation into its mechanism of action and long-term effects on hormonal regulation.

The above studies explored the anti-fertility effects of *R. communis* utilising diverse models ranging from mammalian systems to invertebrate systems and human trials demonstrating both physiological and hormonal mechanisms underlying its contraceptive potential [99,100,101]. McNeil et al. [99] showed that n-hexane seed extracts significantly suppressed spermatogenesis and motility in male rats, with partial recovery observed over time, indicating reversible male contraceptive effects. Desouky et al. [100] found that *R. communis* extract impaired reproductive capacity in *T. pisana* snails through hormonal disruption and glandular damage, while Iornmube et al. [101] reported effective contraceptive outcomes in women using RICOM-1013-J, associated with significantly elevated prolactin levels. Overall, these findings indicate that *R. communis* exhibits potent and multi-pathway anti-fertility activity, making it a promising candidate for both male and female contraceptive development.

### 6.6. Anti-Hepatotoxic Activity

The hepatoprotective activity of *R. communis* has generated interest due to its possible therapeutic benefits for liver health. The study by Shati [102] investigated the hepatoprotective effects of *R. communis* against cyanide-induced liver damage. The results showed that cyanide administration led to a significant decrease in enzymatic activities: catalase (CAT) decreased from 68.6 ± 2.3 to 24.1 ± 1.8 U/mg protein, superoxide dismutase (SOD) from 12.4 ± 0.9 to 5.2 ± 0.4 U/mg protein, glutathione reductase (GSH-Red) from 3.5 ± 0.3 to 1.1 ± 0.1 U/mg protein, and glutathione peroxidase (GSH-Px) from 15.2 ± 1.2 to 5.8 ± 0.6 U/mg protein. The non-enzymatic glutathione (GSH) level decreased from 5.8 ± 0.4 to 2.3 ± 0.2 μmol/mg protein. Additionally, lipid peroxidation increased, indicated by Thiobarbituric Acid Reactive Substance (TBARS) levels rising from 1.8 ± 0.1 to 5.9 ± 0.5 nmol/mg protein. Liver function markers showed significant increases: aspartic transaminase (AST) from 45.2 ± 3.5 to 136.4 ± 10.2 U/L, alanine transferase (ALT) from 42.3 ± 3.1 to 124.7 ± 8.9 U/L, and total bilirubin from 0.9 ± 0.1 to 3.5 ± 0.3 mg/dL. Serum levels of total cholesterol increased from 120.5 ± 8.2 to 198.3 ± 12.4 mg/dL, total lipids from 5.4 ± 0.3 to 11.6 ± 0.7 g/L, and total protein from 6.2 ± 0.5 to 9.8 ± 0.6 g/dL. However, treatment with *R. communis* extract significantly alleviated these adverse effects, restoring enzymatic activities, reducing lipid peroxidation, and normalizing liver function markers and serum parameters. Moreover, the over-expression of genes related to oxidative stress and inflammation (P53, Bcl-2, IL-4, and IL-12) induced by cyanide toxicity was managed effectively by *R. communis* extract. This study concluded that *R. communis* extract has a promising role in treating cyanide-induced hepatotoxicity.

Additionally, Naveen et al. [103] assessed *R. communis*’s hepatoprotective effect in rats, showing the plant’s possible advantages for liver health. The study demonstrated the hepatoprotective qualities of *R. communis* by evaluating its ability to prevent liver damage using animal models and biochemical testing. The effects of aqueous extract of *R. communis* leaves on hepatotoxicity induced by carbon tetrachloride (CCl4) in rats were investigated. The results revealed that the administration of CCl4 led to elevated levels of aspartate aminotransferase (AST) and alanine transaminase (ALT), indicative of liver damage. However, treatment with the aqueous extract of *R. communis* leaves resulted in a significant decrease in AST and ALT levels compared to the standard group treated with CCl4 alone. For instance, AST levels decreased from 266.7 ± 25.68 (standard group) to 144.5 ± 12.92 (Group III) and 161.7 ± 11.92 (Group V) with treatment with 100 mg/kg and 500 mg/kg of the extract, respectively. Similarly, ALT levels decreased from 86.67 ± 4.47 (Standard group) to 30.67 ± 3.565 (Group III) and 42 ± 2.828 (Group V) with treatment with 100 mg/kg and 500 mg/kg of the extract, respectively. These findings underscore the potential hepatoprotective properties of *R. communis* extract against CCl4-induced liver damage.

The above-mentioned studies investigated the anti-hepatotoxic activity of *R. communis* employing different hepatotoxic models, cyanide-induced and carbon tetrachloride (CCl_4_)-induced, to assess its therapeutic potential in mitigating liver damage [102,103]. Shati [102] demonstrated that *R. communis* extract significantly restored antioxidant enzyme levels, reduced lipid peroxidation, and normalised liver function markers in cyanide-exposed rats, while also modulating stress-related gene expression. Similarly, Naveen et al. [103] reported that aqueous extracts of *R. communis* leaves markedly reduced elevated AST and ALT levels caused by CCl_4_, indicating protective effects on liver function. Overall, these studies affirm that *R. communis* exhibits strong hepatoprotective activity by counteracting oxidative stress and restoring normal liver enzyme levels.

### 6.7. Anticancer Activity

*Ricinus communis* has received considerable attention for its potential anticancer effects. Herawati et al. [104] investigated the cytotoxic effects of crude ricin (CR), derived from *R. communis*, on A549 lung cancer cells compared to cisplatin, a standard cancer drug. CR showed cytotoxicity against A549 cells with an IC_50_ value of 40.94 ppm, higher than that of cisplatin (10.98 ppm). Flow cytometry analysis revealed that CR induced apoptosis in A549 cells in a concentration-dependent manner, with a higher proportion of cells undergoing early apoptosis than necrosis. Western blot analysis confirmed that CR activated caspase-9 and caspase-3, indicating apoptotic cell death. Additionally, CR and cisplatin inhibited A549 cell migration in a concentration- and time-dependent manner. Furthermore, CR inhibited autophagy in A549 cells, as evidenced by decreased expression of LC3-II and Beclin-1 and increased expression of p62 and Atg5, suggesting a potential mechanism for its anticancer activity. These findings suggest that CR has potential as an anticancer agent against lung cancer cells, with apoptotic induction and autophagy inhibition as critical mechanisms.

Furthermore, in a study by Hajrah et al. [10], the gene expression profiling of *R. communis* leaf extract was investigated, shedding light on its effects on mammalian cells. MCF7 cells were subjected to sublethal doses of 10 µg/mL and 50 µg/mL of the extract, followed by RNA-Seq analysis to assess changes in gene expression. Significant alterations in gene expression were observed, with 245 upregulated genes and 134 downregulated genes identified at 10 µg/mL and 168 upregulated genes and 73 downregulated genes at 50 µg/mL, compared to control cells treated with DMSO. The study underscored dose-dependent responses to the extract, revealing a subset of genes consistently modulated across both doses. Cluster analysis further corroborated these findings, highlighting the impact of *Ricinus* extract on the gene expression profile of MCF7 cells. These results contribute to understanding the potential anticancer activity of *Ricinus* extract, suggesting its relevance as a therapeutic agent in cancer treatment.

Additionally, *R. communis* fruit extract could potentially possess antimetastatic potential since it has been reported by Mabasa et al. [58] to decrease breast cancer cell migration, adhesion, and invasiveness. The study investigated the impact of the fruit extract on cancer cell behaviour using cell culture and molecular biology techniques, offering insights into the fruit extract’s antimetastatic processes. The fraction exhibited significant reductions in cell viability for both BUD-8 and MCF-7 cells at concentrations between 300 and 500 µg/mL for BUD-8 and 400 to 500 µg/mL for MCF-7. Furthermore, it inhibited various metastasis-related processes, including cell migration, adhesion, invasiveness, and MMP-2 activity. Additionally, the fraction modulated the expression of essential proteins involved in metastasis and angiogenesis, suggesting its potential as an antimetastatic agent for treating malignant cancers.

Moreover, a study by Majumder et al. [11] investigated the cytotoxic effects of *R. communis* fruit extract (RCFE) on breast cancer cells MCF-7 and MDA-MB-231. Treatment with RCFE significantly increased cytotoxicity in both cell lines in a dose- and time-dependent manner. Specifically, 1 µg/mL of RCFE treatment induced considerable cell death in both MCF-7 and MDA-MB-231 cells. Furthermore, RCFE exhibited cytotoxic specificity against various cancer cell lines, including HER2-positive MDA-MB-453, triple-positive ZR-75-1 breast cancer cells, colon cancer cell line HT-29, and adenocarcinoma cell line A549, while showing minimal effects on regular cell lines HEK293 and mouse embryonic fibroblast (MEF) cells. Additionally, RCFE demonstrated inhibitory effects on the migration, adhesion, and invasion of MCF-7 and MDA-MB-231 cells. The extract significantly inhibited cell migration and adhesion in a dose-dependent manner, with higher concentrations exhibiting more pronounced effects; at 10 µg/mL and 50 µg/mL, RCFE significantly reduced adhesion and migration in MCF-7 cells, with 50 µg/mL almost completely inhibiting migration. RCFE treatment also led to a substantial reduction in invasion capability in both cell lines, with 50 µg/mL reducing invasion by up to 85% in MDA-MB-231. Further analysis revealed that RCFE treatment reduced the expression of metastasis-associated matrix metalloproteinase 2 and 9 (MMP-2 and MMP-9) in MCF-7 and MDA-MB-231 cells in a concentration-dependent manner, with reductions of 48% and 64% at 50 µg/mL and 100 µg/mL, respectively. These findings suggest the potential of RCFE as a promising therapeutic agent for breast cancer treatment due to its cytotoxic effects and inhibition of metastatic properties in breast cancer cells.

Moreover, a study by Loan et al. [105] aimed to enhance the specificity of ricin for cancer treatment by conjugating/encapsulating it with DOTAP/DOPE liposomes, forming ricin–liposome complexes. These complexes were then analysed for their characteristics and evaluated for their effects on SKMEL-28 melanoma cells. The results indicated that the ricin–liposome complexes exhibited even size distribution, with an average size of approximately 340 nm. These complexes demonstrated the ability to penetrate cells via endocytosis, with ricin-liposome3 showing the highest efficacy. Importantly, ricin–liposome3 exhibited significant toxicity against SKMEL-28 cells, with the lowest IC_50_ (62.4 ng/mL) among the tested complexes. Furthermore, at the IC_10_ concentration, ricin–liposome3 induced necrosis and apoptosis in SKMEL-28 cells. Additionally, ricin–liposome3 displayed potent anticancer properties, as evidenced by its ability to significantly reduce the migration, invasion, and tumour formation abilities of SKMEL-28 cells compared to control cells. In conclusion, the study suggests that, despite ricin’s reputation as one of nature’s most poisonous substances, its specificity can be enhanced for treating melanoma and potentially other cancers when utilised in complex forms with liposomes. This research offers promising insights into developing targeted therapies using ricin–liposome complexes.

The studies mentioned above evaluated the anticancer potential of *R. communis* utilising various plant parts (seeds, fruits, and leaves) as well as different types of extracts, with a variety of methodologies like gene expression analysis, cytotoxicity assays, flow cytometry, and nanocarrier systems [10,11,58,104,105]. One of the areas where there is a variation is in the solvents and types of extracts utilised, for example, crude ricin protein, methanolic fruit extracts, and aqueous leaf extracts, each with different polarity, hence selectively extracting different types of bioactive compounds [9,10,11,104]. Polar solvents like methanol are better at extracting hydrophilic compounds like flavonoids and phenolic acids, which are characterised as antioxidants with cytotoxic effects, whereas protein-based extracts like ricin exert different apoptotic mechanisms.

The diversity in solvent polarity and target compounds would be one way, then, of accounting for the disparity in potency as well as the mechanism of action between studies. Ricin, for instance, activates caspases and inhibits autophagy, whereas methanolic fruit extracts downregulate metastasis-associated targets such as MMP-2 as well as MMP-9 [11,104]. Equally, the application of liposome encapsulation in Loan et al. [105] reflects increasing interest in maximising specificity as well as cellular internalisation through drug delivery systems, a more focused as well as efficient therapeutic profile than crude or conventional extracts. Overall, the diversity in solvents of extraction, plant components, and delivery forms not only highlights *R. communis*’s rich anticancer potential but also highlights how necessary it is to associate specific bioactivities observed with extraction methods and chemical properties for more judicious therapeutic development.

### 6.8. Antiparasitic Activity

There are recent studies that affirm that *R. communis*, particularly its seed oil and ricinoleic acid, exhibits profound antiparasitic activity. Muñoz-Sánchez et al. [80] tested three commercial-grade castor oils in their in vitro research on the cysticidal activity against the protozoan parasite *Entamoeba histolytica*, the causative agent of amoebiasis. The experiments treated cultures of cysts with each oil at 10–100 µg/mL concentration for 10 h. Morphological examination showed profound membrane disruption, cytoplasm leakage, and dissolution of cyst wall integrity in a dose-dependent fashion. EC_50_ values reported were 35–50 µg/mL, which fall within the calculated intestinal concentrations with frequent oral administration. The authors credited this activity to the high level of ricinoleic acid, which is cytolytic to the membrane of protozoa by fatty acid-induced micellisation and osmotic imbalance [80].

Moreover, Berhanu et al. [106] analysed the isolation and characterisation of ricinoleic acid and methyl ester anthelmintic activity against *R. communis* seed. Petroleum ether extraction, base hydrolysis, and esterification were carried out to obtain pure fatty acid derivatives, which were screened against *Caenorhabditis elegans* as a nematode model and activity was analysed by worm motility and viability using light microscopy. The agents at 1 mg/mL dose level killed more than 97% in 8 h, with no recovery of surviving worms within 24 h. Molecular docking study revealed strong binding energies of ricinoleic acid to succinate dehydrogenase (−5.41 kcal/mol) and glucose-6-phosphate dehydrogenase (−3.76 kcal/mol), enzymes essential to helminth metabolism. Furthermore, in silico ADME screening also revealed satisfactory oral bioavailability, drug-likeness, and low toxicity risk. The study confirmed that ricinoleic acid has great potential for translation as a plant anthelmintic drug [106].

These studies evaluated *R. communis* antiparasitic activity according to various experimental models for determining the efficacy against protozoan and helminth infections [80,106]. Muñoz-Sánchez et al. [80] demonstrated that pharmaceutical-grade castor oil strongly disrupted. *E. histolytica* cysts in vitro with EC_50_ values of 35–50 µg/mL and induced severe morphological damage after 10 h of incubation. This was attributed to the membrane-disrupting activity of ricinoleic acid, which is the principal fatty acid of castor oil. Similarly, Berhanu et al. [106] isolated ricinoleic acid and methyl ester of ricinoleic acid from petroleum ether seed extract of *R. communis* and reported >97% mortality of *C. elegans* at 1 mg/mL. These findings were supported by molecular docking tests, which proved to have good binding to helminthic enzymes such as succinate dehydrogenase and glucose-6-phosphate dehydrogenase. All these tests establish that *R. communis* is significantly potent against parasites through highly active antiparasitic activity, supplemented by the ability of ricinoleic acid to disrupt parasite metabolism and membrane stability. Table 5 summarises the pharmacological activities and mechanisms of *R. communis* extracts.

## 7. Industrial Application

Castor oil is produced from the seeds of the *R. communis* plant and has a wide range of industrial uses in several industries [12]. Castor oil’s several industrial uses highlight its adaptability, durability, and ongoing significance in contemporary manufacturing processes and environmental stewardship programs [107]. In the industrial realm, castor oil made from seeds is a valuable raw material in many industries, as it is an essential component in producing biodegradable plastics, lubricants, and pharmaceuticals [107]. Sen et al. [108] stated that, due to its exceptional viscosity and lubricating qualities, it is an essential ingredient in the creation of high-performance lubricants and greases that guarantee the seamless functioning of industrial machinery and automotive components, even in the face of harsh temperature and pressure fluctuations.

To further address the growing demand for sustainable materials in packaging, automotive parts, and consumer goods, castor oil derivatives, particularly ricinoleic acid, plays a crucial role in the production of biodegradable plastics and polymers [109,110,111]. The castor oil derivatives are also helpful emulsifiers and surfactants, and they are essential for stabilising solutions in various sectors, from paints and coatings to textiles [110,111]. Furthermore, there is growing momentum behind castor oil’s potential as a sustainable biofuel feedstock, providing a greener substitute for traditional fossil fuels and aiding in the fight against climate change [107].

Castor oil has become a leading bio-renewable feedstock in the chemical industry due to its unique profile of hydroxylated fatty acids [112]. It can be used directly as a polyol in the production of polyurethane and polyester, thus enabling the production of green adhesives, coatings, foams, plastics, and composite resins [113]. An extensive study by Mutlu and Meier [112] investigated castor oil as a chemical platform, demonstrating that its major constituent, ricinoleic acid, can undergo esterification and polymerisation without the use of severe catalysts, hence fulfilling the requirements for green chemistry. Their study emphasised the capability of castor oil to be functionalized at the hydroxyl group as well as unsaturation positions, making it extremely versatile for designed chemical synthesis.

Additionally, Lim et al. [113] prepared waterborne polyurethane dispersions based on modified castor oil, showing that the products exhibited improved elongation-at-break, tensile strength, and thermal stability. This work further demonstrates the capacity of castor oil to perform in challenging applications like flexible coatings and biodegradable films with performance metrics that are comparable to, or even better than, petrochemical counterparts. The prevalence of ricinoleic acid in castor oil enables a broad range of functional group modifications, such as epoxidation, amidation, and sulfation, that are critical for the development of high-performance specialty polymers [111]. Additionally, its non-edible status removes competition with food sources, rendering it an economically and ethically acceptable option for chemical feedstocks [114]. This work also highlights the potential for local castor oil production and processing in developing countries, and hence decentralizing bio-industrial development.

In addition to industrial uses, castor oil derivatives are now found to be widely used in the military sector, especially in the preparation of composite solid rocket propellants [115]. The hydroxyl groups of ricinoleic acid make it easy to achieve reactive plasticizers for enhancing the flexibility, burning rate, and energy efficiency of rocket grains [115]. Ahmad et al. [115] compared thermochemical and mechanical behaviour of plasticizers based on castor oil and found that not only did they improve mechanical flexibility of HTPB-based propellants, but also allowed better control over the decomposition kinetics, a desirable safety feature in an explosive compound.

Kovačević et al. [116] presented recent work on composite propellants which established the fact that castor-based polyols improved mechanical and viscoelastic stability upon being added to HTPB-based propellant formulations. Specifically, the research measured storage modulus, tan delta, and cross-link density and concluded that castor-based additives had raised the structural bonding and reduced thermal cracking risks in combustion. Jia et al. [110] demonstrated the polyol esters made from castor oil were effective in stabilizing PVC structures and retaining elasticity against thermal and oxidative environments [110]. Such stabilizing action is similarly in great demand in high-energy military composites with materials exposed to fluctuating environmental and mechanical environments.

These attributes are due to the chemical cross-linking potential of castor oil derivatives, which enable control over combustion performance and support safe storage [115]. In addition, the renewable and low-toxicity nature of castor oil is in line with current defence-agency goals for greener, safer, and more sustainable materials, as defence agencies increasingly look toward replacements for non-renewable or toxic inputs to ammunition, propulsion, and structural systems [111]. Table 6 summarises the industrial applications of *R. communis* in many industries such as pharmaceutical, agriculture, and cosmetics, thus highlighting the purpose and application of the plant.

## 8. Toxicology

*Ricinus communis* has been extensively researched for its toxicological qualities, i.e., ricin, a poisonous protein found in varied amounts throughout the plant, particularly in the seeds, leaves, and pericarp [118]. Ricin is renowned for its high toxicity and has been linked to poisoning in both humans and animals, with symptoms ranging from gastrointestinal distress to organ failure and, in severe cases, death [118]. The assessment of *R. communis* toxicity, as detailed in the study by Joshua et al. [119], provides crucial insights into the safety profile of extracts derived from its seeds. The study’s findings reveal distinct outcomes between the unfermented and fermented methanol extracts of *R. communis seeds*. Notably, the unfermented extract exhibited no toxicity at doses as high as 5000 mg/kg body weight, confirming its relatively benign nature under controlled conditions. In contrast, the fermented extract demonstrated toxicity at the same dosage level, resulting in mortality among test subjects, likely attributable to increased organic acid content from the fermentation process. These findings underscore the critical role of extraction methods in determining the safety of *R. communis* extracts and highlight the necessity for rigorous toxicity assessments in both medicinal and agricultural contexts.

Furthermore, research evaluating its impact on model species such as *Drosophila melanogaste* by Ai et al. [77] emphasised the potential toxicity of *R. communis*, with the plant’s ethanol extract exhibiting toxic effects. The results showed that the ethanol extract of *R. communis* expressed its high toxicity against the 2nd instar larvae of *Drosophila melanogaste* with the LD_50_ value of 64.63 mg/mL, underlining the need for caution in its usage and handling. Additionally, Bernstein et al. [120] conducted a review of the toxicological effects of *R. communis* in traditional medicine. The review’s findings highlight that castor oil, derived from *R. communis* seeds, has historically been significant in pharmacological applications, notably for inducing labour in over 57.7% of treated women within 24 h at doses such as 59 mL. However, the use resulted in adverse effects such as irregular uterine contractions, gastrointestinal distress (vomiting, diarrhoea), and dehydration, underscoring concerns about its potential toxicity for maternal and foetal health. These findings emphasise the critical need for comprehensive toxicological evaluations to ensure the safety of castor oil ingestion, particularly during pregnancy.

The possible effects of *R. communis* on cattle and animal feeding systems were investigated concerning the toxicological consequences of this plant [121]. Research findings on the nutrient profile and toxicity of the *R. communis* seeds in common livestock species revealed that the defatted seed meal contained 32–48% crude protein and approximately 3200 kcal/kg of gross energy. The seeds had a ricin content of 9.3 mg/g and an RCA (*Ricinus communis* Agglutinin) content of 9.9 mg/g. The meal showed high lectin activity, with agglutination occurring at about 4.70 mg/mL. Various detoxification methods showed differing levels of success, with high pH, moist heating, and microbial techniques being most effective in deactivating ricin, though strategies for allergen detoxification were inconclusive. The study concluded that castor seed could be a valuable feedstuff if detoxified safely, cost-effectively, and efficiently for commercial use.

These results demonstrate the wide range of toxicological consequences associated with *R. communis* and the necessity of thorough evaluations to guarantee the safety of its use in different contexts and to reduce any potential negative impacts on the environment and animal health [18,122].

Given the known toxicity of ricin and the other constituents of *R. communis*, traditional and semi-purified plant extracts are widely utilised in indigenous medicine without reported acute toxicity, suggesting the presence of effective detoxification protocols. Disregarding the extreme toxicity of ricin, heat or aqueous traditional preparation techniques have been found to inactivate or reduce the toxic activity of the protein. Ricin is a heat-labile protein that denatures under moist heat, thereby destabilising its two-chain form, making it unable to bind and inactivate ribosomes [91]. Boiling or roasting of seeds and leaves of *R. communis* used extensively in ethnomedicine depletes ricin bioactivity through disruption of disulfide bonds between A and B chains required for its internalisation in host cells [123]. Aqueous preparations by the traditional decoctions also facilitate detoxification by flushing out water-soluble toxins like ricin and ricinine from plant material, particularly if coupled with the drainage of the liquid [81]. Scientific experiments confirm that ricin content in such preparations is far lower or below detectable levels, and toxicity experiments establish reduced effects in animal models and in vitro systems [124].

Pingale et al. [124], in a study of acute toxicity, gave Swiss mice a freshly prepared aqueous leaf juice of *R. communis* at a single dose of 2000 mg/kg body weight and monitored the mice for 14 days. No death, behavioural alteration, or sign of systemic toxicity occurred, and significant parameters such as food/water consumption, body weight, and cage-side behaviour were within the normal range. This high-dose test was negative for toxicity and suggested that water-soluble toxins such as ricin were either inactivated during preparation or available at subtoxic concentrations due to the thermal and aqueous lability of the protein. This parallels ancient means through which aqueous extraction and heat are used to prepare *Ricinus* drugs, also mirroring their relative safety with proper processing.

Such outcomes support the empirical safety of traditional preparation methods and underscore that not all *Ricinus* products are equally risky, especially when processed through traditionally guided detoxification processes. Table 7 represents the LD_50_ phytochemical values components of *R. communis* in animal models across various exposure pathways.

## 9. Future Directions and Research Gaps

Research on *R. communis* is still developing, and several new areas call for investigation and creativity. One promising direction is to clarify the plant’s epigenetic regulatory mechanisms and how they affect its biochemical profile and therapeutic potential. Gaining knowledge about how environmental stimuli alter *R. communis* gene expression patterns might help improve the quantity and calibre of bioactive chemicals produced. Studying the interactions between the plant and symbiotic and soil microbiota species may also reveal new ways to optimise cultivation methods and reduce environmental stressors, increasing the plant’s resilience and productivity in various agroecosystems.

Notable gaps in knowledge still exist in *R. communis* study, even with significant achievements. The thorough characterisation of its secondary metabolites and their cooperative relationships is one crucial area that merits consideration. Although the pharmacological characteristics of some bioactive chemicals have been thoroughly investigated, the entire range of metabolites found in *R. communis* is still poorly understood. Furthermore, additional research is necessary to determine the precise mechanisms regulating these chemicals’ production, storage, and secretion in various plant tissues. Closing these gaps will help us better understand the biochemical complexity of *R. communis* and make it easier to manipulate metabolic pathways specifically for improved industrial and medicinal uses.

## 10. Conclusions

The culmination of this literature review presents a comprehensive overview of its multifaceted attributes and potential applications. Through a synthesis of diverse studies, key findings reveal the plant’s rich composition of bioactive compounds, including ricin and ricinoleic acid, which underpin its pharmacological significance. Across traditional medicinal practices and modern pharmaceutical research, *R. communis* emerges as a promising source of therapeutic agents with diverse biological activities. From its anti-inflammatory and antimicrobial properties to its potential in cancer treatment, the plant’s bioactive constituents hold promise for addressing various health challenges.

Looking forward, the implications for future research underscore several critical avenues for exploration. Firstly, there is a pressing need to deepen our understanding of the underlying mechanisms driving the pharmacological effects of *R. communis* compounds. Unravelling these mechanisms at the molecular level can not only enhance our knowledge of plant-based therapeutics but also inform the development of targeted pharmaceutical interventions. Moreover, exploring synergistic interactions among the plant’s bioactive components may unveil novel therapeutic combinations with enhanced efficacy and reduced side effects.

Beyond its medicinal applications, the literature review also sheds light on the industrial potential of *R. communis*. The plant’s oil-rich seeds offer a sustainable alternative to conventional resources, from biodiesel production to synthesising biopolymers and surfactants. However, realising the full potential of *R. communis* in industrial applications necessitates further research into optimising cultivation practices, enhancing seed yield, and exploring innovative processing techniques. By addressing these challenges, *R. communis* can contribute to diverse sectors, fostering innovation, sustainability, and economic growth.

## Figures and Tables

**Table 1:** Geographical distribution of *Ricinus communis* globally.

Country	Climate	Soil type	References
Argentina	Temperate	Sandy or loamy soil	[65]
Brazil	Tropical and subtropical	Sandy or clay soil	[66]
China	Temperate and subtropical	Loamy soil	[67]
Egypt	Arid and subtropical	Sandy or clay soil	[68]
Ethiopia	Tropical and subtropical	Sandy or clay soil	[69]
India	Tropical	Alluvial, red, and black soil	[70]
Mexico	Tropical and subtropical	Various soil types	[71]
Nigeria	Tropical	Sandy or loamy soil	[72]
Pakistan	Arid and subtropical	Sandy or loamy soil	[73]
South Africa	Temperate and subtropical	Various soil types	[59]
United States	Temperate	Well-drained soil	[74]

**Table 2:** Distribution of *Ricinus Communis* across provinces of South Africa based on habitat, weather/climate conditions, and soil type.

Province	Habitat (of the Plant)	Soil Type	Climate/ Weather Conditions	References
Eastern Cape	Grasslands, scrublands	Sandy, clay	Variable	[60]
Gauteng	Disturbed areas (roadsides, fields)	Sandy, loamy	Temperate	[62]
Kwa-Zulu Natal	Coastal areas, riverbanks	Sandy, clay	Subtropical	[59,63]
Limpopo	Savanna, woodland	Sandy, loamy	Tropical	[59,63]
Mpumalanga	Savanna, woodland	Sandy, loamy	Subtropical	[63]
Northern Cape	Semi-desert regions	Sandy, rocky	Arid	[61]
Northwest	Grasslands, savanna	Sandy, clay	Arid to semi-arid	[75]
Western Cape	Fynbos biome, well-drained soils	Sandy, loamy	Mediterranean	[59]

**Table 3:** Traditional uses of *Ricinus communis* for the treatment of different ailments and diseases.

Plant Part	Disease/Ailments	Treatment	References
Castor oil	Menstrual pain	Castor oil applied topically over the abdominal region helps relieve menstrual pain.	[81]
	Inflammation	The topical application of castor oil is effective in reducing inflammation.	[81]
	Constipation	Consuming castor oil acts as an osmotic laxative, aiding in relieving constipation.	[76]
	Skin conditions	Applied topically for the treatments of skin conditions such as dermatitis, eczema, and acne	[4]
	Intestinal parasites	Ingested orally, typically warm or with herbs to expel intestinal worms and treat parasitic infections like *Strongyloides* and amoebiasis	[79,80]
Leaves	Milk production	Leaves were fed to cattle to increase milk production and applied to women’s breasts for milk production.	[78,81]
	Stomach ache	Drinking an infusion of castor leaves helps relieve stomach ache.	[78,81]
Root	Jaundice	A hot water extract of dried roots taken orally for jaundice.	[81]
Seeds	Headaches	Applying a paste made from seeds on the forehead helps alleviate headaches.	[82]

**Table 4:** Chemical compounds and active constituents found in different plant parts of *Ricinus communis*.

Plant Part	Chemical Compound	Active Constituent	Reference
Leaves	Phenol	Gallic acid, ellagic acid, quercetin	[84,85,87]
	Flavonoid	Rutin, epicatechin, isoquercetin	[84,85,87]
	Alkaloids	Ricinine	[84,85]
Seeds (Fruits)	Flavonoid	Kaempferol	[11,86]
	Tannin	Tannins	[11]
	Fatty acids	Ricin, ricinoleic acid	[2]
	Alkaloid	Ricinine	[2,11,86]
Stems	Tannin	Tannins	[4]
	Terpenoid	β-caryophyllene	[4]
Roots	Indole derivative	Indole-3-acetic acid	[84]
	Triterpenoid	Lupeol	[86]
Flowers	Flavonoid	Rutin	[3,81]

Quantitative values for the specified substances are omitted due to insufficient or unavailable data in the referenced literature. Subsequent research documenting these concentrations will enhance this table.

**Table 5:** Pharmacological activities and mechanisms of *Ricinus communis* extracts.

Activity	Method	Mechanism	References
Antioxidant	Reducing power assay, DPPH scavenging activity assay, FRAP assay, ABTS scavenging assay	The presence of flavonoids and tannins contributes to antioxidant properties	[85,90,91]
Antimicrobial	Disc diffusion, MIC, Transmission Electron Microscopy	Broad-spectrum antimicrobial properties	[4,92,93,94]
Antidiabetic	Streptozocin-induced diabetic rat model, in vitro tests on insulin signalling pathways and glucose metabolism, analytical techniques for chemical composition	Reduction in blood glucose levels, improvement in insulin sensitivity, potential antidiabetic effects of penta-O-galloyl-β-D-glucose and castor oil	[10,95,96]
Anti-Inflammatory	Evaluation of Nrf2 activation, in vivo and in vitro models, microbiological assays	Activation of Nrf2 to mitigate muscular atrophy, antioxidant chemicals supporting anti-inflammatory activity	[10,97,98]
Anti-fertility	Intraperitoneal administration in male rats, semen analysis, oral administration in women volunteers	Reversible suppression of spermatogenesis and sperm motility, alteration in hormone levels associated with contraceptive effects	[100,101]
Anti-hepatotoxic	Evaluation of antioxidant activity, microbiological assays, toxicity experiments, animal models, biochemical testing	Reduction in oxidative stress, potential for liver protection, prevention of liver damage, hepatoprotective qualities	[102,103]
Anticancer	In vitro and in vivo models, molecular biology and cell culture techniques, histological investigations	Anti-tumour action, targeting of cancer stem cells and signalling pathways, and the potential use of ricin as a therapeutic agent	[10,11,58,104,105]
Antiparasitic	In vitro assay on *Entamoeba histolytica* cysts, in vivo assay on Caenorhabditis elegans, molecular docking, ADME profiling	Ricinoleic acid causes membrane disruption in protozoan cysts and inhibits helminth enzymes (succinate dehydrogenase, G6PD)	[80,106]

**Table 6:** Industrial application of *Ricinus communis* in various industrial sectors.

Plant Constituent	Industrial Sector	Application	Purpose	Reference
Castor Oil	Agriculture	Applied as a bio-based crop protection product	Acts as a natural pesticide, reducing reliance on synthetic chemicals for pest control	[3]
	Biofuel production	Processed into biodiesel	Renewable energy source reduces dependence on fossil fuels	[107]
	Pharmaceuticals	Utilised in laxatives and potential drug delivery	Effective laxative properties, potential for controlled drug release	[89]
	Cosmetics	Added to skincare and haircare formulations	Emollient properties moisturise skin and hair and enhance product texture	[77]
	Lubricant and greases	Formulated into lubricants and greases	High viscosity, withstands high pressures and temperatures	[108]
Ricinoleic acid	Plastic and polymer	Used in the synthesis of biodegradable plastics	Enhances sustainability, reduces environmental impact	[110]
Castor oil derivative	Industrial coatings and adhesives	Formulated into coatings and adhesives	Provides excellent adhesion and film-forming properties, enhances durability and corrosion resistance	[110,117]
	Surfactants and emulsifiers	Serve as surfactants and emulsifiers	Stabilises emulsions enhance the dispersion of substances	[110,117]
	Textile	Used in sizing, dyeing, and finishing processes	It improves the texture, strength, and dyeability of textile fibres, and enhances the overall quality of textile products.	[117]

**Table 7:** LD_50_ values for components of *Ricinus communis* in animal models across various exposure pathways.

Compound	Animal Model	Route of Exposure	Reported LD_50_	References
Ricin	Mouse (BALB/c)	Oral (gavage)	~30 µg/kg	[125]
	Rat	Inhalation	0.24 µg/kg	[126]
	Mouse	Inhalation	0.58 µg/kg	[126]
	Swine	Intramuscular	5–10 µg/kg	[127]
Ricinine	Mouse	Intraperitoneal	340 mg/kg	[128]
*Ricinus* leaf aqueous extract	Mouse (Swiss)	Oral (crude)	No lethality at 2000 mg/kg	[124]

## Data Availability

No new data were created or analyzed in this study. Data sharing is not applicable to this article.
